# Boswellic acid inhibits expression of acid sphingomyelinase in intestinal cells

**DOI:** 10.1186/1476-511X-8-51

**Published:** 2009-12-01

**Authors:** Yao Zhang, Rui-Dong Duan

**Affiliations:** 1 Gastroenterology and Nutrition Lab, Biomedical Center, B11, University of Lund, Lund, Sweden

## Abstract

**Background:**

Boswellic acid is a type of triterpenoids with antiinflammatory and antiproliferative properties. Sphingomyelin metabolism generates multiple lipid signals affecting cell proliferation, inflammation, and apoptosis. Upregulation of acid sphingomyelinase (SMase) has been found in several inflammation-related diseases such as inflammatory bowel diseases, atherosclerosis, and diabetes.

**Methods:**

The present study is to examine the effect of 3-acetyl-11-keto-β-boswellic acids (AKBA), a potent boswellic acid, on acid SMase activity and expression in intestinal cells. Both transformed Caco-2 cells and non-transformed Int407 cells were incubated with AKBA. After incubation, the change of acid SMase activity was assayed biochemically, the enzyme protein was examined by Western blot, and acid SMase mRNA was quantified by qPCR.

**Results:**

We found that AKBA decreased acid SMase activity in both intestinal cell lines in dose and time dependent manners without affecting the secretion of the enzyme to the cell culture medium. The effect of AKBA was more effective in the fetal bovine serum-free culture medium. Among different types of boswellic acid, AKBA was the most potent one. The inhibitory effect on acid SMase activity occurred only in the intact cells but not in cell-free extract in the test tubes. At low concentration, AKBA only decreased the acid SMase activity but not the quantity of the enzyme protein. However, at high concentration, AKBA decreased both the mass of acid SMase protein and the mRNA levels of acid SMase in the cells, as demonstrated by Western blot and qPCR, respectively. Under the concentrations decreasing acid SMase activity, AKBA significantly inhibited cell proliferation.

**Conclusion:**

We identified a novel inhibitory effect of boswellic acids on acid SMase expression, which may have implications in human diseases and health.

## Background

Triterpenoids are compounds belonging to a subgroup of terpenoids and are sterols in nature. They are metabolites in plants, playing roles in respiration, photosynthesis, growth regulation, and chemical defence of the plants [1]. Owning to the findings that triterpenoids have anti-inflammatory and anti-carcinogenic effects on human, the interest in the potential application of triterpenoids in human health and disease is increasing [2,3]. Boswellic acids are triterpenoids that are present in Boswellia serrata, a deciduous tree growing in India and China. The genus Boswellia was also found in other parts of the world [4]. Boswellic acids have been used as herb medicine for thousands of years for treatment of inflammatory diseases, mainly rheumatic arthritis [4]. In the last decade, boswellic acids were shown to also have effects against brain tumours, hepatitis, and inflammatory bowel diseases (IBD) [2,5]. Multiple biological effects of boswellic acids have been identified, such as to inhibit 5-lipoxygenase, leukocyte elastase, nuclear factor kappa-B (NF-κB) and cyclooxygenase [6-8]. We previously showed that boswellic acids stimulated apoptosis by a mechanism depending on caspase 8 but independent on Fas/Fas ligand pathways [9]. Based on the chemical structure, at least 3 types of boswellic acids, i.e. β-boswellic acid (BA), keto-β-boswellic acid (KBA), and 3-acetyl-11-keto-β-boswellic acid (AKBA) have been intensively studied. In many cases, AKBA is the most effective one [9].

Sphingomyelin (SM) is a type of sphingolipids which is present in cell membranes and also dietary products. The hydrolysis of SM is triggered by sphingomyelinase (SMase), which cleaves phosphocholine headgroup and turns SM to ceramide. Ceramide is an important molecule with multiple functions including promoting apoptosis, inhibiting cell proliferation, enhancing inflammatory response, and inducing insulin resistance, depending on the concentrations, cellular locations, and type of tissues [10]. Ceramide can be further degraded by ceramidase to sphingosine, which in turn is phosphorylated to sphingosine-1-phosphate (S1P). S1P is a potent molecule that stimulates cell proliferation, migration, and inflammation [11]. The divergent effects of sphingolipids and their metabolites have been summarized in several review articles [10,12,13].

Based on the optimal pH, at least three types of SMase, i.e. neutral, alkaline, and acid SMases have been cloned. Neutral SMase is mainly localized on the plasma membrane and is activated by several cytokines such as TNF-α, and IL-6 [14]. Alkaline SMase is specifically expressed in the intestinal tract playing important roles in digestion of dietary SM and potentially in preventing colonic tumorigenesis [15]. Abnormal forms of alkaline SMase have been identified in human colon and liver cancers [15,16]. Acid SMase is a ubiquitous enzyme wildly expressed in various cell types as a lysosomal enzyme, which hydrolyses SM that is internalized by endocytosis. Recent studies identified that the acid SMase gene (SMPD1) can give rise to two forms of enzyme, one is in the lysosomal and the other is secreted [17]. Upon stimulation, acid SMase can be translocated to the plasma membrane and hydrolyses membrane SM to generate ceramide. Accumulating evidence indicates that acid SMase may have important implications in human health because upregulation of acid SMase has been identified in several diseases such as IBD, atherosclerosis, and diabetes, as reviewed recently [18]. The present study is to investigate the effects of boswellic acids, mainly AKBA, on acid SMase expression in the intestinal cells.

## Methods

### Materials

Caco-2 cells and Int407 cells were obtained from American Tissue Culture Collection (Rockville, MD). BA, ABA (3-acetyl-β-boswellic acid), KBA, and AKBA (purity 98% by HPLC) were obtained from Sabinsa Corporation (Piscataway, NJ, USA). The reagent 4- [3-(4-iodophenyl)-2-(4-nitrophenyl)-2H-5-tetrazoliol]-1, 3-benzene disulfonate (WST-1) for cell proliferation assay was obtained from Roche Diagnostics GmbH (Mannheim, Germany). SM was purified from bovine milk and the choline headgroup was labelled with [^14^C-CH_3_] ([^14^C]-SM). Antibody against acid SMase was purchased from Santa Cruz (Falkenberg, Sweden). The primers for qRT-PCR were purchased from DNA Technology A/S (Risskov, Denmark). All cell culture media and other chemical agents used were purchased from Sigma-Aldrich (Stockholm, Sweden).

### Cell culture

Caco-2 cells were cultured in DMEM with 2 mM glutamine and 4500 mg/l glucose. Int407 cells were cultured in BME with 2 mM L-glutamine. Both media contained 100 IU/ml penicillin, 10 μg/ml streptomycin, and 10% heat inactivated foetal bovine serum (FBS). The cells were incubated at 37°C in an incubator containing 95% air and 5% CO_2_. AKBA and other boswellic acids were dissolved in ethanol as a stock and stored at -20°C. When it was added in the medium, the final concentration of ethanol in the medium was 0.1%, and the medium containing 0.1% ethanol was used as a control. Caco-2 and Int407 cells were cultured to 80% confluence and incubated with AKBA at different concentrations for 24 h. One aliquot of the medium was saved for assaying SMase secreted. The cells were scraped, lysed, sonicated, and centrifuged as described previously [9]. The supernatant was used for SMase assay and total protein determination. For time course study, the cells were incubated with AKBA (50 μM) for 6, 12, 24, and 48 h. At each time point, the cells in both control and experimental groups were lysed and acid SMase activity and cellular proteins were determined. To study the effects of AKBA on the cells in the FBS-free medium, after removing the conventional cell culture medium, the cells were washed with PBS twice, and incubated in FBS-free medium with AKBA.

### Acid sphingomyelinase activity

Acid SMase activity was assayed in 50 mM Tris-maleate buffer pH 5.0 containing 0.15 M NaCl and 0.12% Triton X100 [19]. For each determination, 5 μl sample was added to 95 μl buffer containing 80 pmole [^14^C] SM (~8000 dpm) and incubated at 37°C for 30 min. The reaction was stopped by addition of 0.4 ml chloroform/methanol (2:1, v/v) followed by centrifugation at 10000 g for 5 sec. A portion of the upper phase was taken and the production of [^14^C] phosphocholine was determined by liquid scintillation. The activities of neutral and alkaline SMase were assayed in the similar way with different optimal pH [19]. The protein concentration was determined with Bio-Rad DC Protein Assay Kit using albumin as a standard. The SMase activities were adjusted with the protein concentrations in the samples and expressed as percentage of the values in control group.

To investigate whether AKBA has a direct effect on SMase activity, 30 μl of cell free extract from Caco-2 cells was incubated with AKBA at 0, 25, 50, and 100 μM at 37°C for 1 h. The enzyme activities were determined. The changes of enzyme activity were expressed as percentage of the control (0 concentration of AKBA).

### Western blot analysis

Fifty micrograms of proteins in cell lysate were subjected to 7.5% SDS PAGE and then transferred to nitrocellulose membrane electrophoretically overnight. After blocking the membrane with 5% non-fat dry milk for 2 h, the membrane was probed with anti-acid SMase antibody (1:200) for 1.5 h, and then with donkey anti-goat antibody (1:50000) conjugated with horseradish peroxidase for 1.5 h. The specific acid SMase bands were identified by ECL advance reagents and the remitted light was recorded on Kodak X-ray film. The whole procedure followed the instructions of the manufacturer. The membranes were then stripped and re-probed with anti-β-actin antibody as a loading control.

### RNA isolation and real time reverse transcription PCR

The mRNA levels of acid SMase were quantified by real time reverse transcription PCR (qRT-PCR). Total RNA in the cells treated with AKBA was extracted using TRIzol reagents (Invitrogen, Carlsbad, CA) and the cDNA was generated using 1 μg of total RNA, oligo (dT)_18 _primer, and a cDNA Synthesis Kit (Fermentas, Glen Burnie, MD) according to the instruction of the manufacturer. QRT-PCR was performed on Bio-Rad iCycler system. The reaction volume was 20 μl, including 10 μl of iQ™ SYBR Green Supermix (Bio-Rad), 5 μl of diluted cDNA template (1:500), and 0.3 μl of 20 μM primers. The PCR was initiated at 95°C for 3 min to activate the polymerase, followed by 40 cycles of 95°C for 15 sec, and 60°C for 15 sec. Finally the melting curve analysis was performed to confirm that a single product was amplified and no dimmer was interfered with the reaction. Reactions were performed in triplicate. The cycle threshold (Ct) was analyzed automatically by the iQ™5 Optical System Software (Bio-Rad). Relative expression of acid SMase mRNA was normalized to the level of an internal control gene (GAPDH) by using the 2^ΔΔCT ^method. The primers used in this study were as follows: GAPDH (forward, 5'CATGAGAAGTATGACAACAGCCT3'; reverse, 5' AGTCCTTCCACGATACCAAAGT3') and acid SMase (forward, 5'TGGCTCTATGAAGCGATGGC3'; reverse, 5'TTGAGAGAGATGAGGCGGAGAC3').

### Cell proliferation assay

Cell proliferation was assayed by determination of the cleavage of tetrazolium salt (WST-1) to formazan by mitochondrial dehydrogenase. The amount of formazan formed is proportional to the viable cell numbers. The cells (2×10^4^) were cultured in a 96-well plate and stimulated with AKBA of different concentrations for 24 h. WST-1 was then added in each well. After incubation for 1 h, the production of formazan was read in a microplate reader (Bio-Rad) at 405 nm against 620 nm as a background. The results were expressed as percentage of the control.

### Statistical analysis

Each experiment was conducted at least in triplicate in at least three separate experiments. Statistical analysis was performed by using Student's t-test and one-way ANOVA. Data are presented as mean ± standard error and p < 0.05 was considered statistically significant.

## Results

### AKBA decreased acid SMase activity in Caco-2 cells

**Figure 1:**
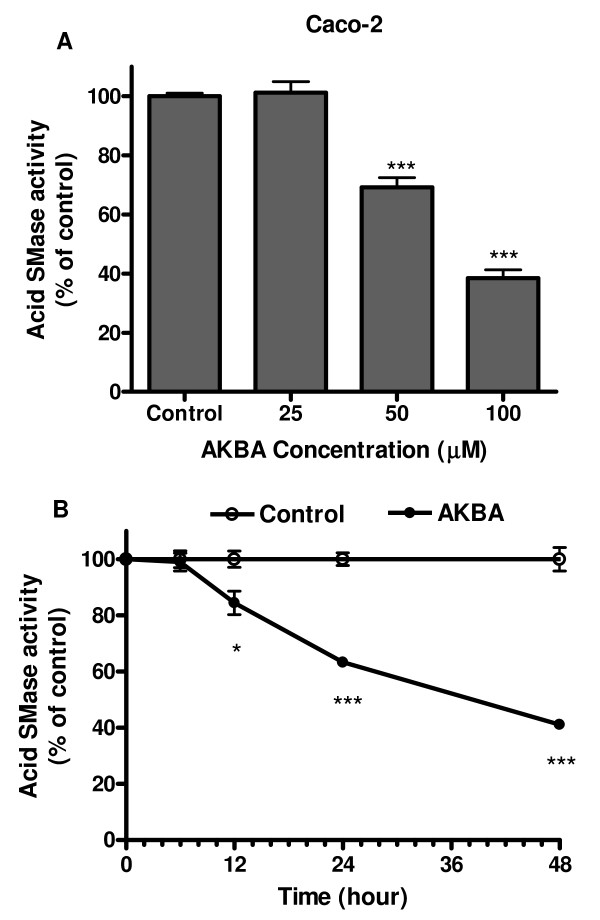
**Changes of acid SMase activity in Caco-2 cells treated with AKBA**. (A): The cells were treated with AKBA in different concentrations for 24 h and acid SMase activities in cell lysate were determined. (B): The cells were treated with AKBA (50 μM) or without AKBA as control for different times. After treatment, the enzyme activity was determined in comparison with the control group at each time point. The activities were adjusted to protein levels in the cells and expressed as percentage of the control. Results are mean ± standard error from 3 separated experiments. * P < 0.05, **P < 0.01, and *** P < 0.001 compared with the control.

After culturing Caco-2 cells with AKBA, the acid SMase activities in the cells were decreased in a dose-dependent manner (Figure 1A). The effects occurred at concentrations of AKBA higher than 25 μM. About 31% inhibition was induced by 50 μM AKBA and 62% inhibition was induced by 100 μM AKBA. No similar changes were identified for either neutral or alkaline SMase activity after AKBA stimulation (data not shown).

The time course of AKBA (50 μM)-induced inhibition of acid SMase was shown in panel B of Figure 1. The acid SMase activity was decreased by 16%, 37%, and 59%, when the cells were treated with AKBA for 12, 24, and 48 h, respectively.

**Figure 2:**
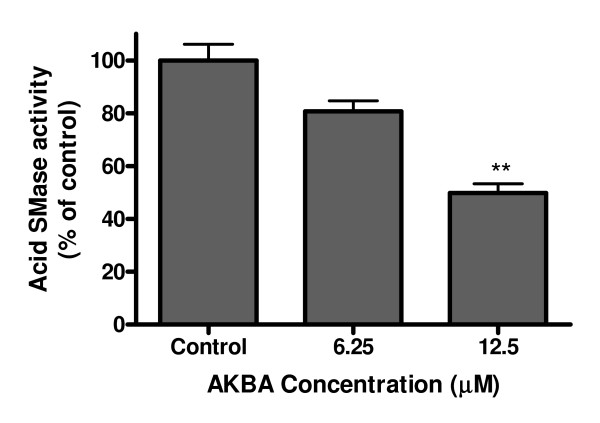
**Effects of AKBA on Caco-2 cells cultured in FBS-free medium**. The cells were cultured conventionally to 80% confluence and then treated with AKBA in DMEM without FBS. Acid SMase activities in cell lysate were determined. The activities in control group were taken as 100%. **P < 0.01 compared with the control.

We also performed an experiment in which Caco-2 cells were incubated with AKBA in the medium without FBS. We found that under this condition, 20% decrease of acid SMase activity could be induced by AKBA at even 6.25 μM (P = 0.058) and 50% decrease by AKBA at 12.5 μM (P = 0.002) (Figure 2). Because culturing the cells in medium without FBS allows the measurement of the released acid SMase [17], we measured the acid SMase activity in this medium after AKBA treatment. No significant change of acid SMase activity induced by AKBA was identified. Neither acid SMase activity was increased in the normal medium with FBS (data not shown).

**Figure 3:**
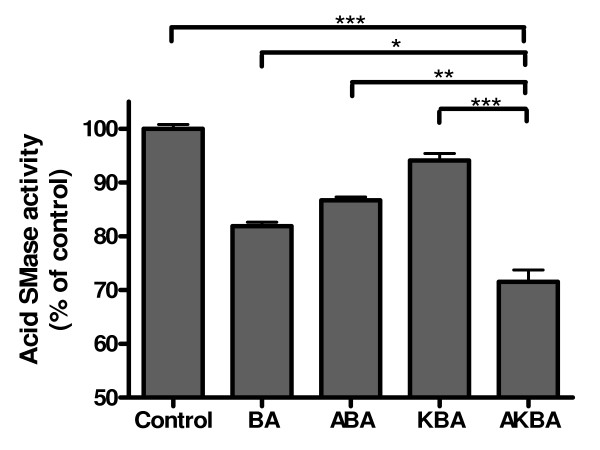
**Comparison of the efficacy of different boswellic acids**. Caco-2 cells were treated with 50 μM BA, ABA, KBA, or AKBA for 24 h. Acid SMase activities in cell lysate were determined. The activities in control group were taken as 100%. Results are mean ± standard error from 3 separated experiments. The statistic significance was analyzed by one-way ANOVA followed by Tukey test. The statistic significances were indicated in the figure. * P < 0.05, ** P < 0.01, and *** P < 0.001.

To compare the efficacy of different boswellic acids, we treated Caco-2 cells with different boswellic acids including BA, ABA, KBA, and AKBA at the concentration of 50 μM for 24 h. All the boswellic acids tested reduced the acid SMase activity and AKBA was the most effective one (Figure 3).

### AKBA also decreased acid sphingomyelinase activity in Int407 cells

**Figure 4:**
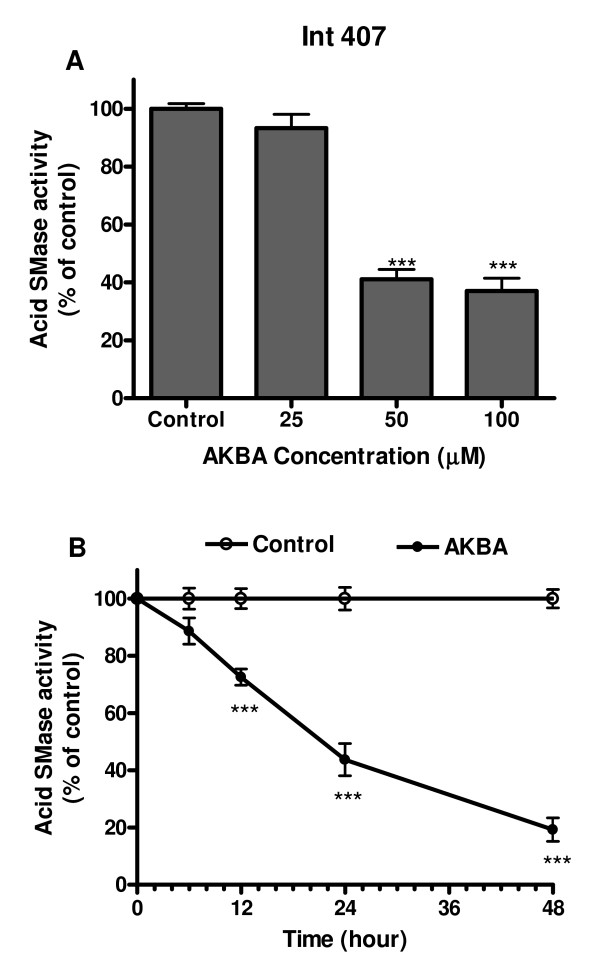
**Changes of acid SMase activity in Int407 cells treated with AKBA**. (A): Non-transformed Int407 cells were treated with AKBA in different concentrations for 24 h and acid SMase activities in the cell lysate were determined. (B): The cells were treated with AKBA (50 μm) or without AKBA as control for different times. After treatment, the enzyme activity was determined in comparison with the control group at each time point. The activities in control group were taken as 100%. Results are mean ± standard error from 3 separated experiments. *** P < 0.001 compared with the control.

To examine whether AKBA also influences acid SMase activity in nontransformed cells, we treated Int407 cells with AKBA at different concentrations or for different times. The results were similar in general to those obtained from the studies with Caco-2 cells (Figure 4), although the Int407 cells appeared to be more sensitive to AKBA than Caco-2 cells, if we compare both the dose and time dependent curves in this figure with Figure 1.

### Mechanism underlying the reduced acid SMase activity by AKBA

To identify the mechanism behind the reduced acid SMase activity by AKBA, we first examined whether AKBA could directly affect the acid SMase activity. We incubated the non-stimulated cell lysate in test tubes with AKBA up to 100 μM at 37°C for 1 h followed by acid SMase assay. Such a treatment did not reduce the acid SMase activity in the lysate (data not shown), suggesting that the reduction of acid SMase activity by AKBA occurs only in the intact cells and may not be a direct inhibition of AKBA on the enzyme protein.

**Figure 5:**
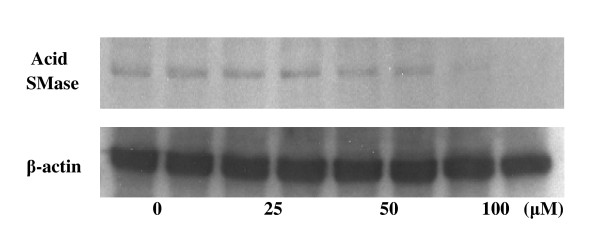
**Western blot showing the effect of AKBA on acid SMase expression**. Caco-2 cells were treated with AKBA in different concentrations for 24 h. Fifty micrograms of proteins were subjected to 7.5% SDS-PAGE and transferred to nitrocellulose membrane electrophoretically. The membrane was probed with anti-acid SMase antibody first and then a secondary antibody. The bands were identified by ECL advance reagents (upper panel). In the lower panel, the membrane was stripped and re-probed with anti-β-actin antibody. The results from two separate samples for each concentration group are presented. Similar results were obtained from other two experiments.

To examine whether the reduced acid SMase activity was associated with changes of the enzyme protein, Western blot was performed. After treating Caco-2 cells with AKBA in different concentrations, we found no significant reduction of the acid SMase mass by AKBA up to 50 μM. However, 100 μM AKBA significantly decreased acid SMase levels as shown in Figure 5. Such a reduction was not caused by a loading error, as similar levels of β-actin were present in all samples.

**Figure 6:**
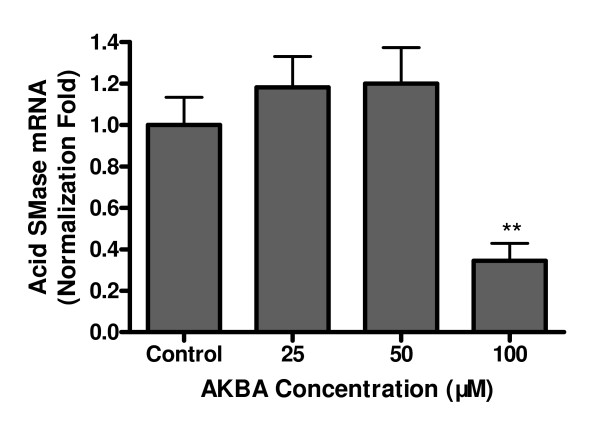
**Changes of acid SMase mRNA in Caco-2 cells after AKBA stimulation**. Total RNA was extracted from Caco-2 cells treated with AKBA and subjected to qRT-PCR to measure the expression of acid SMase mRNA. GAPDH was used as a reference gene. Ct value and relative levels of mRNA were calculated by the iQ™5 Optical System Software. Similar results were obtained from other two experiments. **P < 0.01.

To further understand the mechanism underlying the changed acid SMase protein, real time RT-PCR was performed. We found that the mRNA of acid SMase was not significantly changed in cells treated with AKBA up to 50 μM, but a significant reduction (P < 0.01) of acid SMase mRNA was found in cells treated with 100 μM AKBA (Figure 6).

### AKBA inhibited cell proliferation in Caco-2 cells

**Figure 7:**
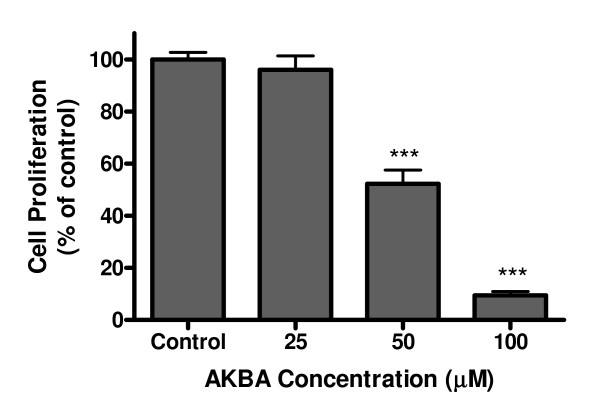
**Effects of AKBA on cell proliferation**. Caco-2 cells were treated with AKBA in different concentrations for 24 h. Cell proliferation was assayed by the cleavage of WST-1 reagent. The results are expressed as mean ± standard error from 3 separate experiments. *** *P *< 0.001 compared with control.

When the cells were treated with AKBA at the same concentrations used in the experiment, the cell proliferation was inhibited in a dose-dependent manner. AKBA at 50 μM inhibited the cell proliferation by 52%, whereas 100 μM AKBA strongly inhibited the cell proliferation by 90% (Figure 7).

## Discussion

In the present study we identified a novel inhibitory effect of boswellic acids on acid SMase activity in cell culture. The effect was dose-dependent. About 30% inhibition could be induced by 50 μM AKBA in the presence of FBS and by 6~12 μM AKBA in the absence of FBS. The increased efficacy of AKBA in the absence of FBS indicates a protective effect of serum against AKBA. The reduction of acid SMase activity occurred 12 h after incubation and was generally similar in transformed intestinal Caco-2 cells and non transformed Int407 cells.

The reduced acid SMase activity was not caused by a direct inhibition of AKBA on acid SMase protein, because in vitro incubation of AKBA with cell free extract did not reduce the enzyme activity. Since acid SMase gene can give rise to two forms of the enzyme, one in the lysosomes and the other secreted [17], we also examined whether the reduced enzyme activity in the cell extract was due to an increased secretion of the enzyme. By simultaneous measuring of the activity in the cell free extract and medium with or without serum, we did not find obvious secretion of acid SMase to the medium before and after AKBA stimulation. Since previous finding of a secreted acid SMase was mainly in studies with endothelial cells [18], the failure to identify secretion of acid SMase in the intestinal cells may indicate a cell type specificity. Although acid SMase was found in the intestinal lumen previously, the activity is likely derived from the shedding of the intestinal mucosal cells [20].

To identify the mechanism underlying the decreased acid SMase activity in the cells, both Western blot and qPCR were performed. We found that at 50 μM, AKBA did not affect the contents of acid SMase protein and acid SMase mRNA, although the enzyme activity was decreased about 30%. The results indicate that at this concentration, the reduced activity was not caused by an inhibition of gene transcription and translation, but presumably by an interference of post-translational modification or maturation of the enzyme. It has been demonstrated that post-translational modifications of acid SMase involve glycosylation, phosphorylation, and proteolytic processing, which are important for maturation of the enzyme [18]. AKBA has been shown to affect various protein kinases such as CDK4 [21], PI3K [22,23], IKK [8], and MAPK [22], and also some types of phosphatase [24] in various cell types including the intestinal cells. Whether these effects could affect acid SMase activity is of interest for further investigation. However, at high concentration, AKBA inhibited the expression of acid SMase at the transcriptional levels, as convincingly demonstrated by the results of Western blot and qPCR.

The finding that AKBA inhibits both activity and expression of acid SMase in the intestinal cells may have implications in the intestinal diseases, particularly IBD. Previous studies in animal models showed that AKBA inhibited the IBD induced by indomethacin and decreased the mucosal damage, with a mechanism linked to a decrease in leukocyte-endothelial cell interaction [25]. Our study indicates an additional mechanism for AKBA to inhibit the intestinal inflammation. Recently Sakata et al reported that acid SMase might play important roles in promoting the development of IBD, because inhibition of acid SMase resulted in significantly improvements of the inflammation in both animal and cell culture studies [26]. In agreement with the concept, treating intestinal cells with exogenous SMase from bacteria was recently shown to induce the damage of the intestinal barrier functions [27]. In supporting the hypothesis, our study also showed that AKBA inhibited cell proliferation at the concentrations inhibiting acid SMase activity.

In addition, the inhibition of acid SMase activity by AKBA may have implications in other diseases. Ceramide generated from hydrolysis of SM was previously considered in general an important molecule that inhibits cell proliferation and induces apoptosis. This concept has been changed as many studies showed that the effects of ceramide are more complicated varying with both locations and ways to be generated [12]. Ceramide generated by acid SMase in several cases has been shown to be proinflammatory and may stimulate cell proliferation. Several inflammatory factors such as TNF-α and LPS are able to stimulate acid SMase expression [28]. Increased acid SMase expression has been found in various diseases that threaten human health, such as atherosclerosis, heart failure, insulin resistance, cystic fibrosis, lung inflammation, and peritonitis as recently reviewed [18]. In many cases, inhibition of acid SMase improved the pathogenesis of the diseases. Studies with acid SMase -/- mice also support pathogenic effects of acid SMase in some diseases mentioned above [29-31].

Based on the literatures above, AKBA might potentially be a candidate of a new type of antiinflammatory drugs targeting acid SMase. It is stable and safe. However, the absorption of boswellic acids after oral administration is relatively poor and the absorbed boswellic acids are accumulated in the intestinal mucosal cells [32]. This could favour its potential utilization against intestinal diseases such as IBD, as it may increase the exposure of colon to AKBA. On the other hand, pharmacokinetic studies showed that AKBA in plasma did not undergo phase 1 metabolism [33], whether it can be given intravenously in a relatively low dose is an interesting question for investigation.

## Conclusion

We identified a novel inhibitory effect of AKBA on acid SMase expression in intestinal cells, which may have implications in human diseases and health.

## Competing interests

The authors declare that they have no competing interests.

## Authors' contributions

YZ was involved in all bench work, data acquisition, analysis and interpretation and manuscript preparation. RDD participated in the design of the study, data analysis and manuscript preparation. All authors read and approved the final manuscript.
